# Efficacy of Pirfenidone vs. Placebo in Unclassifiable Interstitial Lung Disease, by Surgical Lung Biopsy Status: Data From a *post-hoc* Analysis

**DOI:** 10.3389/fmed.2022.897102

**Published:** 2022-06-17

**Authors:** Maria Molina-Molina, Michael Kreuter, Vincent Cottin, Tamera J. Corte, Frank Gilberg, Klaus-Uwe Kirchgaessler, Judit Axmann, Toby M. Maher

**Affiliations:** ^1^ILD Unit, Respiratory Department, University Hospital of Bellvitge, Institut d'Investigació Biomèdica de Bellvitge (IDIBELL), L'Hospitalet de Llobregat, Barcelona, Spain; ^2^Centro de Investigación Biomédica en Red Enfermedades Respiratorias (CIBERES), Madrid, Spain; ^3^Center for Interstitial and Rare Lung Diseases, Dep-Pneumonology, Thoraxklinik, University of Heidelberg, Heidelberg, Germany; ^4^German Center for Lung Research, Heidelberg, Germany; ^5^National Reference Coordinating Center for Rare Pulmonary Diseases, Louis Pradel Hospital, Hospices Civils de Lyon, Member of ERN-LUNG, Lyon, France; ^6^IVPC, Université Claude Bernard Lyon 1, UMR754, INRAE, Lyon, France; ^7^Department of Respiratory Medicine, Royal Prince Alfred Hospital, University of Sydney, Sydney, NSW, Australia; ^8^Medical School, University of Sydney, Camperdown, NSW, Australia; ^9^F. Hoffmann-La Roche, Ltd., Basel, Switzerland; ^10^Interstitial Lung Disease Unit, Royal Brompton Hospital, London, United Kingdom; ^11^National Heart and Lung Institute, Imperial College London, London, United Kingdom; ^12^Hastings Center for Pulmonary Research and Division of Pulmonary, Critical Care and Sleep Medicine, Keck School of Medicine, University of Southern California, Los Angeles, CA, United States

**Keywords:** lung function, pirfenidone, *post-hoc* analysis, surgical lung biopsy, unclassifiable interstitial lung disease

## Abstract

Approximately 12–13% of patients with interstitial lung disease (ILD) are diagnosed with unclassifiable ILD (uILD), often despite thorough evaluation. A recent Phase 2 study (NCT03099187) described a significant effect of pirfenidone vs. placebo on forced vital capacity (FVC) measured by site spirometry in patients with progressive fibrosing uILD (hereafter referred to as the pirfenidone in uILD study). Here, we present the results from a *post-hoc* analysis of this study to assess patient baseline characteristics and the efficacy of pirfenidone vs. placebo analyzed by surgical lung biopsy (SLB) status. Mean FVC (mL) change over 24 weeks was included as a *post-hoc* efficacy outcome. Of 253 randomized patients, 88 (34.8%) had a SLB and 165 (65.2%) did not. Baseline characteristics were generally similar between SLB subgroups; however, patients who had a SLB were slightly younger and had a higher 6-min walk distance than those without a SLB. Mean FVC change over 24 weeks for pirfenidone vs. placebo was −90.9 vs. −146.3 mL, respectively, in patients who had a SLB, and 8.2 vs. −85.3 mL, respectively, in patients without a SLB. Overall, the results from the *post-hoc* analysis identified that pirfenidone may be an effective treatment in progressive fibrosing uILD over 24 weeks, irrespective of SLB status; however, caution should be taken when interpreting these data due to several limitations. There are differences in the treatment effect of pirfenidone between the subgroups that require further pathological and radiological investigation. In this manuscript, we also descriptively compared baseline characteristics from the overall pirfenidone in uILD study population with other uILD populations reported in the literature, with the aim of understanding if there are any similarities or differences within these cohorts. Most baseline characteristics for patients in the pirfenidone in uILD study were within the ranges reported in the literature; however, ranges were wide, highlighting the heterogeneity of uILD populations.

**Clinical Trial Registration:**

ClinicalTrials.gov, identifier: NCT03099187.

## Introduction

Interstitial lung diseases (ILDs) are a diverse, heterogeneous group of respiratory diseases characterized by fibrosis and/or inflammation of the lung (1). Achieving a diagnosis of a specific ILD is important to inform prognosis and guide treatment decisions (1–3); however, it is a complex process that requires detailed assessment of clinical, radiological, and pathological features by a multidisciplinary team (MDT) (2, 3). Despite thorough evaluation by a MDT, which for some patients may also include undergoing a surgical lung biopsy (SLB), a specific diagnosis cannot be achieved in ~12–13% of patients with ILD (4), and in these cases, patients are defined as having unclassifiable ILD (uILD).

There are many factors that may contribute to the uncertainty around achieving a specific ILD diagnosis, including: the inability to perform a SLB due to age, disease severity, other comorbidities, or the patient not being willing to have a SLB; SLBs that are inadequate or insufficient for diagnosis; or discrepancies between clinical, radiological, and/or pathological features (5–8). Ultimately, patients receive a diagnosis of uILD when all other possible causes have been excluded, rather than due to identification of uILD-specific features. Moreover, different centers and clinicians may apply different diagnostic thresholds or carry out varying intensities of diagnostic investigations. Therefore, uILD populations can be heterogeneous, which makes studying this patient population challenging. In an attempt to study a well-defined uILD population, a recent Phase 2 trial assessed the efficacy and safety of pirfenidone vs. placebo in patients with progressive fibrosing uILD over 24 weeks (hereafter referred to as the pirfenidone in uILD study; NCT03099187) (9). In this study, the primary endpoint was mean predicted change in forced vital capacity (FVC) from baseline over 24 weeks, measured by daily home spirometry. Analysis of the primary endpoint was not possible due to technical issues with home spirometry, and application of the pre-specified linear regression model was not suitable in patients with a small number of readings collected over a short period of time. However, the results from key secondary and exploratory outcomes suggested that pirfenidone may be an effective treatment for patients with progressive fibrosing uILD. Additionally, the safety profile of pirfenidone was comparable with that in patients with IPF (10), and no new safety signals were identified (9).

Here, we present the results of a *post-hoc* analysis from the pirfenidone in uILD study, in which baseline characteristics and the efficacy of pirfenidone vs. placebo were analyzed by the presence or absence of a SLB in patients with progressive fibrosing uILD over 24 weeks. Additionally, we compared baseline patient demographics and characteristics from the pirfenidone in uILD study with data published from other uILD populations, with the aim of highlighting any similarities or differences in the cohorts.

## Materials and Methods

### Study Design and Patient Population

The pirfenidone in uILD study was a multicenter, international, double-blind, randomized, placebo-controlled, 24-week, Phase 2 trial (NCT03099187) in patients with progressive fibrosing uILD. Eligible patients were aged between 18 and 85 years; had percent predicted FVC of ≥45%; had percent predicted carbon monoxide diffusing capacity (DLco) of ≥30%; had >10% fibrosis on high-resolution computed tomography; had a high-resolution computed tomography scan from the previous 12 months; and had a diagnosis of progressive fibrosing uILD. Fibrosing uILD was defined as a fibrosing ILD that could not be given a specific ILD diagnosis with moderate or high confidence following MDT evaluation at each participating center. Therefore, eligible patients had either a diagnosis of uILD or a low-confidence diagnosis of a specific ILD. Progressive disease was defined as either >5% absolute decline in percent predicted FVC or significant symptomatic worsening not due to cardiac, pulmonary (except worsening of underlying uILD), vascular, or other causes (as determined by the investigator) within the previous 6 months. Full details regarding the methods of this study have been previously reported (9, 11).

### *Post-hoc* Analysis

Baseline characteristics were analyzed in a *post-hoc* analysis by SLB status (patients who had a SLB and patients who did not have a SLB). *Post-hoc* efficacy endpoints were evaluated over 24 weeks in patients who had a SLB and patients who did not have a SLB. These *post-hoc* efficacy endpoints were: change in FVC (mL) over 24 weeks, measured by site spirometry; the percentage of patients who experienced a >5% or >10% decline in percent predicted FVC over 24 weeks, measured by site spirometry; the proportion of patients who experienced a >15% categorical decline in percent predicted DLco over 24 weeks; and the proportion of patients who experienced a >50 m decline in 6-min walk distance (6MWD) over 24 weeks.

#### Statistical Analyses

*Post-hoc* efficacy endpoints were assessed in the intent-to-treat population. Change in FVC (mL) was estimated using a linear regression model. Two-sided confidence intervals (CIs) for the mean value were based on the percentiles of the t-distribution. For change in FVC (mL), only patients with at least 2 post-baseline measurements were included, and all data from baseline to Week 24 were used without imputation of values for patients who discontinued early. A Cochran–Mantel–Haenszel test was used to analyze categorical changes in percent predicted FVC between treatment groups. A logistic regression model was used to compare categorical changes in percent predicted DLco and 6MWD between treatment groups.

### Literature Search for Comparison of uILD Patient Populations

To identify other populations of patients with uILD in the literature, references were collected through searches of PubMed on April 7, 2021, by use of the terms “unclassified interstitial lung disease,” “unclassifiable interstitial lung disease,” “unclassified ILD,” “unclassifiable ILD,” “uILD”, “unclassified idiopathic interstitial pneumonia,” “unclassifiable idiopathic interstitial pneumonia,” “unclassified IIP,” “unclassifiable IIP,” “undefined ILD,” and “undetermined ILD.” Some searches used these terms in the title, others in the title or abstract. Deeper searches included the terms “unclassified interstitial lung disease,” “unclassifiable interstitial lung disease,” “unclassified ILD,” “unclassifiable ILD,” “uILD,” “unclassified idiopathic interstitial pneumonia,” and “unclassifiable idiopathic interstitial pneumonia” alongside “pirfenidone” or “nintedanib” in the title or abstract. No limits were placed on date; however, only articles in English were included. Abstracts resulting from these searches were reviewed and relevant references were obtained. References that included baseline characteristics for patients with uILD were selected for inclusion. These data were compared with patient baseline characteristics from the overall pirfenidone uILD study population and were summarized descriptively.

## Results

### *Post-hoc* Analysis in the Pirfenidone in uILD Study

#### Baseline Demographics and Characteristics Analyzed by SLB Status

Between May 2017 and June 2018, 253 patients were randomized to pirfenidone (*n* = 127) or placebo (*n* = 126) (9). Of these patients, 88 (34.8%) patients had a SLB and 165 (65.2%) did not have a SLB. The majority of patients had a diagnosis of uILD without any features suggestive of another form of ILD, regardless of SLB status. The proportion of patients in each subgroup with low-confidence diagnoses of other ILDs (i.e., other diagnoses were considered but could not be given with moderate or high confidence following MDT evaluation) are reported in Table 1. The mean time from diagnosis to randomization was generally similar in patients regardless of SLB status, except for patients who had a SLB and received pirfenidone (37.2 months vs. 25.5–27.6 months).

**Table 1:** Demographics and baseline characteristics of patients in the pirfenidone in uILD study analyzed by SLB status.

Characteristic^*^	**Patients with historical SLB** **(*****n*** **=** **88)**		**Patients without historical SLB** **(*****n*** **=** **165)**	
	**Pirfenidone** **(*n* = 40)**	**Placebo** **(*n* = 48)**	**Pirfenidone** **(*n* = 87)**	**Placebo** **(*n* = 78)**
Age at screening, years	64.4 (9.6)	63.3 (10.6)	69.7 (9.9)	70.3 (7.0)
Male, *n* (%)	27 (67.5)	25 (52.1)	43 (49.4)	44 (56.4)
White, *n* (%)	38 (95.0)	48 (100.0)	82 (94.3)	75 (96.2)
BMI, kg/m^2^	31.2 (6.5)	29.5 (5.1)	29.5 (5.9)	29.3 (5.5)
Percent predicted FVC, %	75.5 (18.7)	73.9 (20.1)	73.3 (18.9)	74.0 (20.0)
Percent predicted DLco, %	48.8 (12.3)	50.4 (12.6)	45.0 (12.4)^‡^	49.1 (14.8)^§^
Percent predicted FEV_1_, %	77.1 (17.2)	77.4 (19.4)	76.5 (19.1)	78.5 (21.7)
FEV_1_:FVC ratio	0.81 (0.06)	0.84 (0.07)	0.83 (0.07)	0.83 (0.06)
6MWD, m	437.0 (116.3)	425.7 (98.9)	370.8 (108.7)	374.5 (109.5)
Time from ILD diagnosis to randomization, months	37.2 (50.1)	26.6 (38.4)	25.5 (32.8)	27.6 (33.3)
Diagnosis of uILD, *n* (%)				
Low-confidence RA-ILD	0	0	0	0
Low-confidence SSc-ILD	0	1 (2.1)	0	0
Low-confidence undifferentiated CTD-ILD	0	1 (2.1)	3 (3.4)	1 (1.3)
Low-confidence cHP-ILD	2 (5.0)	3 (6.3)	8 (9.2)	6 (7.7)
Low-confidence idiopathic NSIP-ILD	0	0	4 (4.6)	3 (3.8)
Low-confidence sarcoidosis-ILD	0	0	0	0
Low-confidence myositis-ILD	0	0	0	0
Low-confidence other defined ILD	0	0	1 (1.1)	0
Fulfills criteria of IPAF	3 (7.5)	8 (16.7)	13 (14.9)	10 (12.8)
uILD^¶^	35 (87.5)	35 (72.9)	58 (66.7)	58 (74.4)

* *Data are presented as mean (SD) unless otherwise specified*.

‡ *n = 86*.

§ *n = 77*.

¶ *Diagnosis of uILD without any features suggestive of another form of ILD*.

*6MWD, 6-minute walk distance; BMI, body mass index; cHP, chronic hypersensitivity pneumonitis; CTD, connective tissue disease; DLco, diffusing capacity of the lung for carbon monoxide; FEV_1_, forced expiratory volume in 1 second; FVC, forced vital capacity; ILD, interstitial lung disease; IPAF, interstitial pneumonia with autoimmune features; NSIP, non-specific interstitial pneumonia; RA, rheumatoid arthritis; SD, standard deviation; SLB, surgical lung biopsy; SSc, systemic sclerosis; uILD, unclassifiable interstitial lung disease*.

Baseline characteristics were generally similar regardless of SLB status; however, some differences were observed between the subgroups. Patients who had a SLB and received either pirfenidone or placebo were younger and walked further in the 6-min walk test than patients who did not have a SLB and received either pirfenidone or placebo (Table 1).

#### Efficacy of Pirfenidone vs. Placebo Over 24 Weeks Analyzed by SLB Status

Efficacy data for pirfenidone vs. placebo are presented in Table 2. At Week 24, the mean change in FVC measured by site spirometry in patients who had a SLB and received either pirfenidone or placebo was −90.9 and −146.3 mL, respectively (treatment difference: 55.4 mL; 95% CI −52.6, 163.4; Table 2). In patients who did not have a SLB and received either pirfenidone or placebo, mean change in FVC over 24 weeks measured by site spirometry was 8.2 and −85.3 mL, respectively (treatment difference: 93.5 mL; 95% CI 15.2, 171.9; Table 2).

**Table 2:** Summary of *post-hoc* efficacy endpoints to week 24 in patients in the pirfenidone in uILD study analyzed by SLB status.

**Parameter**	**Patients with historical SLB (*****n*** **=** **88)**			**Patients without historical SLB (*****n*** **=** **165)**		
	**Pirfenidone** **(*n* = 40)**	**Placebo** **(*n* = 48)**	**Pirfenidone vs. placebo**	**Pirfenidone** **(*n* = 87)**	**Placebo** **(*n* = 78)**	**Pirfenidone vs. placebo**
**Predicted FVC change from baseline to week 24 measured by site spirometry, mL**						
Mean (95% CI)^*^	−90.9^‡^ (−179.9, −2.0)	−146.3^§^ (−213.8, −78.9)	55.4 (−52.6, 163.4)	8.2^¶^ (−47.9, 64.4)	−85.3^|^ (−140.8, −29.8)	93.5 (15.2, 171.9)
**FVC change from baseline to week 24 measured by site spirometry, % predicted**						
Patients with >5% decline, *n* (%)^**^	20 (50.0)	28 (58.3)	–	27 (31.0)	45 (57.7)	–
Odds ratio (95% CI)	–	–	0.7 (0.3, 1.6)	–	–	0.3 (0.2, 0.6)
Patients with >10% decline, *n* (%)^**^	8 (20.0)	14 (29.2)	–	10 (11.5)	19 (24.4)	–
Odds ratio (95% CI)	–	–	0.6 (0.2, 1.6)	–	–	0.4 (0.2, 1.0)
**DLco change from baseline to week 24, % predicted**						
Patients with >15% decline, *n* (%)^‡‡^	2 (5.0)	5 (10.4)	–	0	7 (9.0)	–
Odds ratio (95% CI)	–	–	0.5 (0.1, 2.5)	–	–	n.c. (n.c., n.c.)
**6MWD change from baseline to week 24, m**						
Patients with decline >50 m, *n* (%)^‡‡^	9 (22.5)	12 (25.0)	–	25 (28.7)	23 (29.5)	–
Odds ratio (95% CI)	–	–	0.9 (0.3, 2.3)	–	–	1.0 (0.5, 1.9)

* *Estimated from linear regression model. Only patients with at least two post-baseline measurements were included in the analysis. Two-sided CI for mean value was based on percentiles of the t-distribution*.

‡ *n = 38*.

§ *n = 46*.

¶ *n = 76*.

| *n = 72*.

** *Treatment comparison was analyzed using a Cochran–Mantel–Haenszel test stratified by randomization stratification factors*.

‡‡ *Treatment comparison was analyzed using a logistic regression*.

*6MWD, 6-minute walk distance; CI, confidence interval; DLco, diffusing capacity of the lung for carbon monoxide; FVC, forced vital capacity; n.c., not calculable; SLB, surgical lung biopsy; uILD, unclassifiable interstitial lung disease*.

In patients who had a SLB and received either pirfenidone or placebo, the proportion of patients who experienced a categorical decline in percent predicted FVC >5% over 24 weeks was 50.0% and 58.3%, respectively [odds ratio (OR) 0.7 (95% CI 0.3, 1.6)], and the proportion of patients who experienced a categorical decline in percent predicted FVC >10% over 24 weeks was 20.0% and 29.2%, respectively [OR 0.6 (95% CI 0.2, 1.6); Table 2]. In patients who did not have a SLB and received either pirfenidone or placebo, the proportion of patients who experienced a categorical decline in percent predicted FVC >5% over 24 weeks was 31.0 and 57.7%, respectively [OR 0.3 (95% CI 0.2, 0.6)], and the proportion of patients who experienced a categorical decline in percent predicted FVC >10% over 24 weeks was 11.5 and 24.4%, respectively [OR 0.4 (95% CI 0.2, 1.0); Table 2].

### Comparison of Baseline Demographics and Characteristics From the Pirfenidone in uILD Study Population With Other uILD Populations

Baseline characteristics for the overall pirfenidone in uILD study population (*n* = 253) are presented in Supplementary Table 1, and a comparison of baseline characteristics from this study with those from other uILD populations is presented in Supplementary Table 2.

In total, 189 publications were initially identified in the literature search. Of these, 10 relevant publications from 2018 to 2021 that have reported demographics and baseline characteristics for other uILD patient populations were selected for inclusion (4, 12–20). Of these publications, one study included patients enrolled in a clinical trial (19), eight prospective or retrospective studies included patients in real-world cohorts (12–18, 20), and one publication described a meta-analysis of 22 prospective and retrospective studies (4). Publications that did not report demographics and baseline characteristics, that were included in the meta-analysis, or that were published prior to the meta-analysis were excluded.

Across these uILD patient populations, the majority of patients received a diagnosis of uILD following evaluation by a MDT (Supplementary Table 2). Many studies did not report SLB rates; however, in those that did, there were differences in the reported rates. In the pirfenidone in uILD study, 34.8% of patients had a SLB, whereas in other uILD populations, the reported SLB rates ranged from 0% to 100% (4, 12, 14–18). Similarly, several studies did not present any information regarding patients with uILD who met the criteria for interstitial pneumonia with autoimmune features (IPAF), and in the studies that did report this information, the number of patients who met the criteria for IPAF varied (4, 13, 16, 17). In the pirfenidone in uILD study, 13.4% of patients fulfilled the criteria for IPAF; however, in other uILD populations, the proportion of patients who fulfilled the criteria for IPAF ranged from 2.1% to 25.0% (4, 13, 16, 17). At baseline, age and body mass index were generally similar in this study compared with the other uILD populations; however, a wide range of mean ages was reported across the other populations (53.0–68.4 years).

Baseline percent predicted FVC, percent predicted DLco, and percent predicted forced expiratory volume in 1 second (FEV_1_) for the overall population in this study were within the range of values reported for other uILD populations. However, it should be noted that a wide range of values was reported for each measure across the studies (mean FVC: 67.8–84.9%; mean DLco: 43.8–65%; mean FEV_1_: 69.9–86.3%). The median baseline 6MWD reported for patients in the overall study population was lower than that reported for the other uILD patient populations (387.0 m vs. 435 m and 442 m; Supplementary Table 2).

## Discussion

The purpose of the analyses presented here were to: (1) assess baseline characteristics and the efficacy of pirfenidone vs. placebo, analyzed by the presence or absence of a SLB in patients with progressive fibrosing uILD over 24 weeks; and (2) compare baseline patient demographics and characteristics from the pirfenidone in uILD study with other uILD populations to highlight any similarities or differences in the cohorts.

Results from the *post-hoc* analysis of data from the pirfenidone in uILD study identified that demographics and baseline characteristics were generally similar between patients in the pirfenidone in uILD study who had a SLB and those who did not have a SLB; however, noteworthy differences were apparent. Patients who had a SLB were slightly younger and walked further during the 6-min walk test than patients who did not have a SLB, which is in line with expectations given that SLB can often be impracticable in many patients due to age and disease severity (8). These findings are comparable to those of a previous study, which reported that patients with uILD who had a SLB were slightly younger than those who did not have a SLB (21). Interestingly, in the previous study, baseline percent predicted FVC and DLco were lower in patients who had a SLB than in patients who did not have a SLB (21); however, this was not observed in the *post-hoc* analysis population. Furthermore, patients who had a SLB and received pirfenidone had a longer mean time from diagnosis to randomization than patients who did not have a SLB and received pirfenidone, and whilst this might be expected, it may be a confounding factor.

Additionally, the results of this *post-hoc* analysis revealed that outcomes over 24 weeks were similar in patients who received placebo regardless of SLB status, with the exception of mean change in FVC measured by site spirometry, which was greater in patients who had a SLB and received placebo than in those who did not. However, it is not possible to draw any conclusions regarding disease trajectory from these data due to factors including differences in patient numbers between subgroups and any potential differences in disease status (e.g., stable vs. progressive) between patients prior to enrollment. However, it may be interesting for future studies to investigate this further.

In terms of response to pirfenidone treatment, the results of this *post-hoc* analysis expand on the primary findings from the pirfenidone in uILD study. Although analysis of the primary endpoint was not possible, secondary endpoints indicated that pirfenidone may be an effective treatment in progressive fibrosing uILD over 24 weeks (9), and similar results were observed in the *post-hoc* analysis. Indeed, irrespective of SLB status, the mean change in FVC from baseline and the proportions of patients who experienced a categorical decline of >5% or >10% in percent predicted FVC over 24 weeks were numerically lower in patients who received pirfenidone compared with placebo. Additionally, the proportion of patients who had a SLB and experienced a categorical decline of >15% in percent predicted DLco was numerically lower in patients who received pirfenidone compared with placebo. These results suggest that SLB status does not substantially impact this treatment benefit; however, further studies in larger patient populations are required to determine if the differences between pirfenidone and placebo are statistically significant in patients with and without a SLB. It could be argued that a treatment effect in patients without a SLB could be explained by patients in this group being more likely to have IPF-like disease because their histopathological pattern is unknown. Therefore, the group without a SLB would possibly include some patients with histopathological usual interstitial pneumonia, whilst in the group who had a SLB, those patients with histopathological usual interstitial pneumonia would likely have been allocated a diagnosis of IPF and would no longer be classified as uILD. The fact that the treatment benefit was not impacted by SLB status indicates that the treatment effect of pirfenidone may not simply be due to efficacy in a subgroup of patients who may have an unidentified IPF-like pathological pattern. As noted above, the disease status of patients prior to enrollment (e.g., stable vs. progressive) may also potentially influence the effect of pirfenidone in this study; however, the relevant data to determine if this was the case in this study are not available. Therefore, there were differences in the treatment effect of pirfenidone between the subgroups that require further pathological and radiological investigation.

Data on the safety and tolerability of pirfenidone are not presented in this manuscript, however, it should be noted that no meaningful differences were observed between patients who had an SLB and patients who did not have an SLB.

This is not the only study to have investigated antifibrotics in progressive fibrotic ILDs other than IPF. In the INBUILD study of patients with progressive fibrotic ILDs, the annual rate of decline in FVC was significantly lower in patients who received nintedanib than in those who received placebo (22). Moreover, the treatment benefit of nintedanib was maintained in a subgroup analysis of data analyzed by diagnostic category (19). In the RELIEF study of patients with progressive fibrosing ILDs other than IPF (connective tissue disease-associated ILD, fibrotic non-specific interstitial pneumonia, chronic hypersensitivity pneumonitis, and asbestos-induced lung fibrosis), pirfenidone treatment was efficacious vs. placebo; however, it should be noted that the RELIEF study was terminated prematurely due to slow enrollment (23). In summary, the findings from this *post-hoc* analysis add to existing studies in the literature, which have suggested that antifibrotics may have a benefit in ILDs other than IPF.

Despite the study design of the pirfenidone in uILD study resulting in a well-defined uILD population (9), one of the main challenges associated with studying uILD populations is that patients generally receive a diagnosis of uILD when all other ILDs have been excluded; and centers or clinicians may apply varying intensities of diagnostic investigations or different diagnostic thresholds when evaluating a patient. Clinicians may decide against performing a SLB for reasons such as: inability to perform a SLB in patients who are too old or frail; patients declining a SLB; or the benefits not outweighing the risks in some cases of mild or stable diseases (17). In such scenarios, cryobiopsy may be chosen as a safer alternative to SLB (24), or it may be possible to form a working diagnosis without a SLB (8). Based on this, we performed a literature review to compare baseline patient demographics and characteristics from the pirfenidone in uILD study with other uILD populations to determine if there were any similarities or differences in the cohorts. Unsurprisingly, in the papers we identified in the literature, SLB rates varied greatly (4, 12, 14–18). However, it is also important to acknowledge that some studies did not report whether SLBs were performed or not (thus it is not known if SLBs were not performed in these populations at all, or if some patients may have undergone a SLB and it was simply not reported), and in one study, only patients who underwent a SLB were included (12). These observations highlight the differences in the diagnostic investigations performed in uILD patient populations and underline the potential importance of the findings from our *post-hoc* analysis.

Ultimately, differences in diagnostic investigations or thresholds lead to heterogeneity and make it difficult to determine if uILD populations represent a cluster of disorders with distinct demographics and disease trajectories. Indeed, no clear patterns were observed for baseline characteristics across the uILD populations compared here, and for several characteristics, a wide range of baseline values was reported across all studies (4, 9, 12–20). Whilst these findings appear to support the suggestion that patients with uILD represent a heterogeneous population, the uILD populations compared here represent a variety of study designs, including clinical trials and retrospective and prospective real-world studies. Although the observed heterogeneity may be reflective of clinical practice, the impact of differences in study design should also be considered. Furthermore, patients enrolled in the pirfenidone in uILD study were diagnosed with progressive fibrosing uILD, whereas patients in other uILD populations in the literature may or may not have been diagnosed with progressive disease. Moreover, there is no universally accepted definition of progressive disease in non-IPF ILD, and it has been shown that different definitions of progressive disease used across studies can result in meaningful differences in the number of patients diagnosed with progressive disease (25). This highlights that further studies with more robust methodology are required in order to study a well-defined uILD population.

There are several limitations associated with both the *post-hoc* and literature search analyses that should be considered when interpreting these results. For the *post-hoc* analysis, the *post-hoc* nature of this analysis, the small number of patients in some of the subgroups, and the relatively short treatment duration may limit interpretation of the results, and as such, further studies would be required to establish the efficacy of pirfenidone based on SLB status. Despite most patients in the study having a diagnosis of uILD without any features suggestive of another form of ILD, uILD patient populations are heterogeneous and, therefore, it is possible that some patients who did not have a SLB may have had an underlying usual interstitial pneumonia pattern typical of IPF. However, it is important to note that patients with a low-confidence IPF diagnosis were excluded from this study. Another limitation is that patients were not required to have undergone a SLB within a specific time period prior to enrollment, and reasons for patients not undergoing a biopsy were not collected during the pirfenidone in uILD study. Furthermore, data regarding cryobiopsy were not available and it has been shown that cryobiopsy may offer a less invasive alternative to SLB, and had this procedure been available to patients, there may have been more patients who underwent lung biopsy. Additionally, the comparisons between the patient population in the pirfenidone in uILD study and other uILD populations identified in the literature may be limited by differences in study design. As the uILD populations in the literature represent a combination of clinical trials and prospective and retrospective real-world studies, this may have led to distinct patient populations and could impact the data collected. The heterogeneity observed across the uILD populations discussed here, particularly in relation to the definitions of “unclassifiable” and “progressive,” may also impact the interpretation and generalization of the data presented in this manuscript.

## Conclusions

*Post-hoc* analysis of data from the pirfenidone in uILD study identified that pirfenidone may be an effective treatment vs. placebo in progressive fibrosing uILD over 24 weeks, irrespective of SLB status; however, it is not possible to draw definitive conclusions from these data due to several limitations. Further pathological and radiological investigations may be required to further assess the treatment effect of pirfenidone in patients with and without a SLB. Additionally, we identified that baseline characteristics of patients in the pirfenidone in uILD study were generally similar to baseline characteristics for other uILD populations in the literature; however, as expected, the populations were heterogeneous. This heterogeneity may reflect different diagnostic thresholds or practices, and highlights the need for studies with more robust methodology in order to study a well-defined uILD population.

## Data Availability Statement

Qualified researchers may request access to individual patient-level data through the clinical study data request platform (https://vivli.org/). Further details on Roche's criteria for eligible studies are available here (https://vivli.org/members/ourmembers/). For further details on Roche's Global Policy on the Sharing of Clinical Information and how to request access to related clinical study documents, see here (https://www.roche.com/research_and_development/who_we_are_how_we_work/clinical_trials/our_commitment_to_data_sharing.htm).

## Ethics Statement

The trial was conducted in full conformance with the International Conference on Harmonisation E6 guideline for Good Clinical Practice and the principles of the Declaration of Helsinki, or the laws and regulations of the country in which the research was conducted, whichever afforded the greater protection to the individual. Informed consent was obtained from each participant by the study investigator before any study-specific screening procedures were carried out.

## Author Contributions

All authors listed have made a substantial, direct, and intellectual contribution to the work and approved it for publication.

## Funding

This study is sponsored by F. Hoffmann-La Roche, Ltd. This trial was initially developed as part of an NIHR-funded Clinician Scientist Fellowship awarded to TM (NIHR Ref: CS-2013-13-017) and subsequently adapted following discussions with F. Hoffmann-La Roche, Ltd. F. Hoffmann-La Roche were involved in data interpretation and the writing of the manuscript, in collaboration with the academic authors. The corresponding author had full access to all data in the studies and had final responsibility for the decision to submit for publication.

Medical writing support was provided by Leigh Clements, PhD, of CMC AFFINITY, McCann Health Medical Communications, funded by F. Hoffmann-La Roche, Ltd. TM was supported by a NIHR-funded Clinician Scientist Fellowship (NIHR Ref: CS-2013-13-017) and a British Lung Foundation Chair in Respiratory Research (C17-3).

## Conflict of Interest

MM-M or her institution has received grants from AstraZeneca, Boehringer Ingelheim, BRN, Chiesi, Esteve-Teijin Healthcare (ETH), GlaxoSmithKline, InterMune (a fully owned subsidiary of F. Hoffmann-La Roche, Ltd. since 2014), and F. Hoffmann-La Roche, Ltd., outside the submitted work. MK has received fees for speaking and/or organizing education from AstraZeneca, Bayer, Boehringer Ingelheim, Chiesi, ERS, F. Hoffmann-La Roche, Ltd., GlaxoSmithKline, and Novartis; has received fees for consulting from Boehringer Ingelheim, F. Hoffmann-La Roche, Ltd., Galapagos, GlaxoSmithKline, and InterMune (a fully owned subsidiary of F. Hoffmann-La Roche, Ltd. since 2014); and has received research funding, including institutional funding, from BMBF, Boehringer Ingelheim, DFG, the German Center for Lung Research (DZL), F. Hoffmann-La Roche, Ltd., Hopp Stiftung, InterMune (a fully owned subsidiary of F. Hoffmann-La Roche, Ltd. since 2014), Lilly, Lungenfibrose E.V., Medac, Olympus, UKGM, and WATL. VC reports personal fees and non-financial support from Actelion; grants, personal fees, and non-financial support from Boehringer Ingelheim; personal fees from AstraZeneca, Bayer/MSD, Celgene, Galapagos, Galecto, Novartis, Sanofi, and Shionogi; and personal fees and non-financial support from F. Hoffmann-La Roche, Ltd. and Promedior, Inc. (acquired by Roche in February 2020) outside the submitted work. TC has received unrestricted educational grants, travel assistance, and speaker fees from, and served on advisory boards for, Boehringer Ingelheim; has received unrestricted educational grants and speaker fees from, and served on advisory boards for, F. Hoffmann-La Roche, Ltd.; has received unrestricted educational grants from Actelion, Ltd., Bayer, Galapagos, and Sanofi; and has served on advisory boards for AstraZeneca, Bristol Myers Squibb, and Promedior, Inc. (acquired by Roche in February 2020). FG is an employee of F. Hoffmann-La Roche, Ltd. K-UK and JA are employees and shareholders of F. Hoffmann-La Roche, Ltd. TM has, *via* his institution, received industry-academic funding from AstraZeneca and GlaxoSmithKline R&D, and has received consultancy or speaker fees from AstraZeneca, Bayer, Blade Therapeutics, Boehringer Ingelheim, Bristol Myers Squibb, F. Hoffmann-La Roche, Ltd., Galapagos, Galecto, GlaxoSmithKline R&D, IQVIA, Pliant, Respivant, and Theravance.

## Publisher's Note

All claims expressed in this article are solely those of the authors and do not necessarily represent those of their affiliated organizations, or those of the publisher, the editors and the reviewers. Any product that may be evaluated in this article, or claim that may be made by its manufacturer, is not guaranteed or endorsed by the publisher.

## Abbreviations

6MWD: 6-minute walk distance
BMI: body mass index
cHP: chronic hypersensitivity pneumonitis
CI: confidence interval
CTD: connective tissue disease
DLco: diffusing capacity of the lung for carbon monoxide
FEV_1_: forced expiratory volume in 1 second
FVC: forced vital capacity
ILD: interstitial lung disease
IPAF: interstitial pneumonia with autoimmune features
IQR: interquartile range
MDT: multidisciplinary team
n. c.: not calculable
NR: not reported
NSIP: non-specific interstitial pneumonia
OR: odds ratio
PMX-DHP: direct hemoperfusion with a polymyxin B-immobilized fiber column
RA: rheumatoid arthritis
SD: standard deviation
SLB: surgical lung biopsy
SOC: standard of care
SSc: systemic sclerosis
uILD: unclassifiable interstitial lung disease.
