# Supplementary material for: Efficacy of Pirfenidone vs. Placebo in Unclassifiable Interstitial Lung Disease, by Surgical Lung Biopsy Status: Data From a post-hoc Analysis
Source: Front Med (Lausanne). 2022 Jun 17;9:897102. doi: 10.3389/fmed.2022.897102 (PMC9247211; doi:10.3389/fmed.2022.897102)
Supplement: Supplementary file 1 [file Data_Sheet_1.PDF]

SUPPLEMENTARY MATERIAL

Supplementary Table 1 Demographics and baseline characteristics of the pirfenidone in uILD study population.

**Characteristic***	**Treatment group**		
	**Pirfenidone (*n* = 127)**	**Placebo (*n* = 126)**	**Overall population (*N* = 253)**
Age at screening, years	68.0 (10.1)	67.7 (9.2)	67.8 (9.6)
Male, *n* (%)	70 (55.1)	69 (54.8)	139 (54.9)
White, *n* (%)	120 (94.5)	123 (97.6)	243 (96.0)
BMI, kg/m^2	30.1 (6.1)	29.4 (5.3)	29.7 (5.7)
Percent predicted FVC	74.0 (18.8)	74.0 (20.0)	74.0 (19.4)
Percent predicted DLco	46.2 (12.4)[‡]	49.6 (13.9)[§]	47.9 (13.3)[¶]
Percent predicted FEV_1	76.7 (18.4)	78.1 (20.8)	77.4 (19.6)
FEV_1:FVC ratio	0.82 (0.07)	0.83 (0.06)	0.83 (0.06)
6MWD, m	391.6 (114.9)	394.0 (108.1)	392.8 (111.4)
Time from ILD diagnosis to randomization, months	29.2 (39.3)	27.2 (35.2)	28.2 (37.3)
Historical SLB, *n* (%)	40 (31.5)	48 (38.1)	88 (34.8)
Time from most recent SLB to randomization, months	25.5 (43.7)	30.1 (36.6)	28.0 (39.8)
Diagnosis of uILD, *n* (%)			
Low-confidence RA-ILD	0	0	0
Low-confidence SSc-ILD	0	1 (0.8)	1 (0.4)
Low-confidence undifferentiated CTD-ILD	3 (2.4)	2 (1.6)	5 (2.0)

Low-confidence cHP-ILD	10 (7.9)	9 (7.1)	19 (7.5)
Low-confidence idiopathic NSIP-ILD	4 (3.1)	3 (2.4)	7 (2.8)
Low-confidence sarcoidosis-ILD	0	0	0
Low-confidence myositis-ILD	0	0	0
Low-confidence other defined ILD	1 (0.8)	0	1 (0.4)
Fulfils criteria of IPAF	16 (12.6)[‖]	18 (14.3)	34 (13.4)
uILD**	93 (73.2)	93 (73.8)	186 (73.5)

*Data are presented as mean (SD) unless otherwise specified.

[‡]*n* = 126.

[§]*n* = 125.

[¶]*n* = 251.

[‖]This value is different to the primary publication (9) due to mis-stratification of one patient.

**Diagnosis of uILD without any features suggestive of another form of ILD.

6MWD, 6-minute walk distance; BMI, body mass index; cHP, chronic hypersensitivity pneumonitis; CTD, connective tissue disease; DLco, diffusing capacity of the lung for carbon monoxide; FEV_1, forced expiratory volume in 1 second; FVC, forced vital capacity; ILD, interstitial lung disease; IPAF, interstitial pneumonia with autoimmune features; NSIP, non-specific interstitial pneumonia; RA, rheumatoid arthritis; SD, standard deviation; SLB, surgical lung biopsy; SSc, systemic sclerosis; uILD, unclassifiable interstitial lung disease.

Supplementary Table 2 Comparison of demographics and baseline characteristics from other uILD populations.

**Characteristic***	**Pirfenidone in uILD study population (*N* = 253) (9)**	**Wells AU, et al. 2020 (*n* = 114) (19)**	**Guler SA, et al. 2018 (*n* = 1,060) (4)**	**Traila D, et al. 2018 (*n* = 27) (18)**	**Fisher JH, et al. 2019 (*n* = 286) (16)**	**Krauss E, et al. 2020 (*n* = 140) (17)**	**Fang C, et al. 2020 (*n* = 40) (15)**	**Cherchi R, et al. 2020 (*n* = 99) (12)**	**Wong AW, et al. 2020 (*n* = 343) (20)**	**Enomoto N, et al. 2020[‡§] (*n* = 24) (14)**	**Davidsen JR, et al. 2021[¶] (*n* = 141) (13)**
Study design	Clinical trial; primary analysis	Clinical trial; subgroup analyses	Meta-analysis of 22 prospective and retrospective studies	Real-world study; retrospective	Real-world study; prospective	Real-world study; retrospective	Real-world study; retrospective	Real-world study; retrospective	Real-world study; prospective	Real-world study; retrospective	Real-world study; prospective
Treatment permitted, yes/no	Yes; pirfenidone, placebo	Yes; nintedanib, placebo	NR	NR	Yes; SOC	Yes; SOC	Yes; pirfenidone	NR	Yes; SOC	Yes; SOC, PMX-DHP	NR
Age, years	67.8 (9.6)	68.4 (9.4)	65.9 (63.2, 68.9)[‖]	53.0 (14.6)	67.6 (10.5)	68.2 (11.0)	59.4 (8.6)	66 (10)	65 (12)	79.5 (64–85)	69 (60–74)
Male, *n* (%)	139 (54.9)	62 (54)	51.9 (45.1, 58.6)[‖]	9 (33.3)	150 (52.5)	81 (57.9)	21 (52.5)	73 (74)	185 (54)	13 (54.2)	86 (61.0)
BMI, kg/m^2	29.7 (5.7)	NR	NR	26.6 (5.4)	30.3 (5.8)	24.2 (4.8)	23.2 (2.3)	27.0 (4.7)	30 (6)	NR	NR
Percent predicted FVC, %	74.0 (19.4)	69.8 (15.4)	72.0 (68.3, 75.8)[‖]	73.4 (22.7)	76.0 (20.7)	67.8 (22.1)	73.2 (19.1)	84.9 (25.5)	81 (20)	62.0 (42.1–95.7)**	87.1 (77.8–102.0)
Percent predicted DLco, %	47.9 (13.3)[‡‡]	45.2 (11.9)	47.3 (42.1, 52.4)[‖]	47.4 (16.1)	59.2 (19.5)	43.8 (26.3)	50.0 (16.0)	59.6 (18.3)	65 (21)	65.5 (44.7–90.2)**	57.0 (40.0–64.0)
Percent predicted FEV$_1$, %	77.4 (19.6)	NR	NR	69.9 (18.3)	NR	76.3 (55.6)	NR	86.3 (23.1)	NR	NR	89.0 (76.0–98.0)
6MWD, m	387.0 (314.5–480.0)[¶]	NR	NR	NR	NR	367.8 (121.9)	NR	NR	NR	442 (320–578)**	435 (99.0–520.0)
MDT, yes/no	Yes	NR	Variable[§§]	Yes	Yes	Yes	Yes[¶¶]	Yes	Yes	NR	Yes

SLB, *n* (%)	88 (34.8)	NR	4.9–100	4 (15)	65 (22.7)	105 (75)	0	99 (100)‖	NR	7 (29.2)	NR***
Fulfills criteria of IPAF, *n* (%)	34 (13.4)	NR	18.0–25.0	NR	47 (16)	6 (4.3)	NR	NR	NR	NR	3 (2.1)

*Data are presented as mean (SD) unless otherwise specified.
‡Patients with acute exacerbation of unclassifiable idiopathic interstitial pneumonia were included in this study.
§Data are presented as median (range).
¶Data are presented as median (IQR)
|Data from this meta-analysis are presented as weighted mean (95% CI). The weighted mean (by the number of unclassifiable ILD cases reported in each study) was estimated by random-effects model to account for across-study heterogeneity.
**Data were collected within 12 months before acute exacerbation.
‡‡*n* = 251.
§§Eleven of the 22 studies included in this meta-analysis reported use of an MDT. Importantly, while the other 11 studies did not report use of a MDT, they may still have used a MDT without reporting it.
¶¶Diagnosis was confirmed by two experienced pulmonologists and one radiologist during routine clinical care.
‖Only patients undergoing a SLB were included in this study.
***Patients included in this study were undergoing a transbronchial lung cryobiopsy.
6MWD, 6-minute walk distance; BMI, body mass index; CI, confidence interval; DLco, diffusing capacity of the lung for carbon monoxide; FEV_1, forced expiratory volume in 1 second; FVC, forced vital capacity; ILD, interstitial lung disease; IPAF, interstitial pneumonia with autoimmune features; IQR, interquartile range; MDT, multidisciplinary team; NR, not reported; PMX-DHP, direct hemoperfusion with a polymyxin B-immobilized fiber column; SD, standard deviation; SLB, surgical lung biopsy; SOC, standard of care; uILD, unclassifiable interstitial lung disease.
